# Substitution of the Rev-response element in an HIV-1-based gene delivery system with that of SIVmac239 allows efficient delivery of Rev M10 into T-lymphocytes

**DOI:** 10.1186/1742-6405-5-11

**Published:** 2008-06-05

**Authors:** Narasimhachar Srinivasakumar

**Affiliations:** 1 Division of Hematology/Oncology, Department of Medicine, Vanderbilt University, Nashville, Tennessee, USA

## Abstract

**Background:**

Human immunodeficiency virus type 1 (HIV-1)-based gene delivery systems are popular due to their superior efficiency of transduction of primary cells. However, these systems cannot be readily used for delivery of anti-HIV-1 genes that target constituents of the packaging system itself due to inimical effects on vector titer. Here we describe HIV-1-based packaging systems containing the Rev-response element (RRE), of simian immunodeficiency virus (SIV) in place of the HIV-1 RRE. The SIV RRE-containing packaging systems were used to deliver the anti-Rev gene, Rev M10, into HIV-1 susceptible target cells.

**Results:**

An HIV-1 based packaging system was created using either a 272- or 1045-nucleotide long RRE derived from the molecular clone SIVmac239. The 1045-nucleotide SIV RRE-containing HIV-1 packaging system provided titers comparable to that of the HIV-1 RRE-based one. Moreover, despite the use of HIV-1 Rev for production of vector stocks, this packaging system was found to be relatively refractory to the inhibitory effects of Rev M10. Correspondingly, the SIV RRE-based packaging system provided 34- to 130-fold higher titers than the HIV-1 RRE one when used for packaging a gene transfer vector encoding Rev-M10. Jurkat T-cells, gene modified with Rev M10 encoding HIV-1 vectors, upon challenge with replication defective HIV-1 in single-round infection experiments, showed diminished production of virus particles.

**Conclusion:**

A simple modification of an HIV-1 gene delivery system, namely, replacement of HIV-1 RRE with that of SIV, allowed efficient delivery of Rev M10 transgene into T-cell lines for intracellular immunization against HIV-1 replication.

## Background

Lentivirus-based gene delivery systems have been used extensively for gene transfer into a variety of different target cells, both *ex vivo *and *in vivo *[1]. A possible application of lentivirus-based packaging systems based on human immunodeficiency virus type 1 (HIV-1) is for the delivery of anti-HIV-1 genes, such as siRNAs or genes that encode transdominant proteins, to HIV-1 susceptible cells for intracellular immunization [2]. However, the delivery of such genes using a packaging system based on HIV-1 is hampered by the inhibitory effect of the anti-HIV-1 genes, such as Rev M10, on the expression of either the helper or gene-transfer vector RNAs in the producer cells, resulting in low vector titers. Thus, HIV-1-based packaging systems are most useful if the anti-HIV-1 genes target those regions or products of the viral genome not present in the helper or gene-transfer vector constructs or target host genes, such as the gene for the CCR5 coreceptor [3-5].

All lentivirus-based gene delivery systems contain packaging or helper constructs for expression of viral Gag/Pol and gene transfer vectors that encode the transgene of interest. The expression of RNAs from both the Gag/Pol helper and the gene transfer vector constructs in HIV-1-based packaging systems requires the coexpression of viral trans-acting regulatory protein Rev and its target sequence in the viral envelope coding region, the Rev response element or RRE [6,7].

It was previously shown that HIV-1 Rev could function with the RRE from HIV-2 or simian immunodeficiency virus (SIV), but the Rev proteins from HIV-2 or SIV were unable to function with HIV-1 RRE [8,9]. It should therefore be feasible to replace the HIV-1 RRE with the RRE from SIV in an HIV-1-based packaging system. In the present study, HIV-1 packaging systems containing the SIV RRE from SIVmac239 were created and found to provide titers equivalent to those obtained with HIV-1 Rev/RRE-based system. Additionally, despite the use of HIV-1 Rev for vector stock production, the SIV RRE-based HIV-1 packaging system was found to be relatively refractory to the inhibitory effects Rev M10, a transdominant mutant of Rev [10]. The SIV RRE containing HIV-1 packaging system was used for the delivery of Rev M10 to Jurkat T-cells, which, upon challenge with HIV-1 in single-round infection assays, produced fewer virus particles than untransduced control cells.

## Results

### Effect of homologous and heterologous transport proteins on vector production by HIV-1 and SIV RRE-based HIV-1 packaging systems

**Figure 1:**
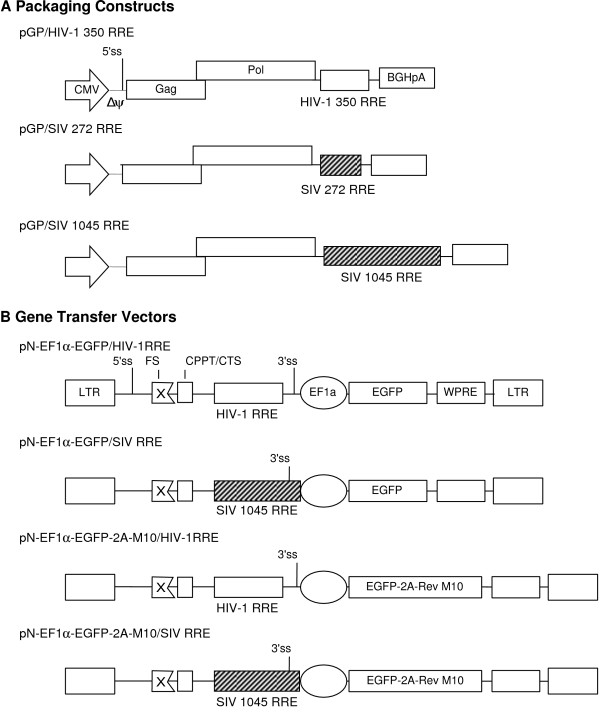
**Schematic representation of HIV-1 packaging and gene transfer vector constructs containing HIV-1 or SIV RRE**. A) The packaging constructs contain Gag/Pol coding sequence derived from pNL4-3 inserted downstream of the human cytomegalovirus (CMV) immediate early promoter in pCDNA3. The RRE from HIV-1 (350 nt) or from SIVmac239 (272 or 1045 nt) was positioned downstream of the Gag/Pol coding sequence. Polyadenylyation sequence in pCDNA3 is derived from bovine growth hormone gene (BGHpA). B) The gene transfer vectors were derived from pNL4-3 and contain a transgene expression cassette consisting of Elongation factor 1 alpha promoter/enhancer elements (EF1α) driving the enhanced green fluorescent protein (EGFP) or a fusion protein consisting of EGFP-2A-Rev M10. Woodchuck post-transcriptional regulatory element (WPRE) was positioned downstream of the transgene. HIV-1 or SIV RRE was present upstream of the transgene expression cassette. Δψ: Deletion in the HIV-1 encapsidation signal between nt 751 and nt 779 of pNL4-3; LTR: HIV-1 long terminal repeat; FS: Frame-shift mutation in *gag*; CPPT/CTS: Central polypurine tract/central termination sequence; 2A: Foot and mouth disease virus 2A cleavage factor; M10: Rev M10; 5'ss: 5' splice site; 3'ss: 3' splice site.

The SIVmac239 RRE exhibits about 87% homology with HIV-2 RRE. Lewis and coworkers mapped the RRE within HIV-2 *env *[9] and showed that the RRE activity was localized within a 1045 bp fragment. The activity could be narrowed down to a smaller fragment of 272 bp. Since SIV RRE had not been tested in an HIV-1 packaging system, several packaging and gene transfer vectors containing HIV-1 or SIV RREs were created (Figure 1). The packaging constructs contained either a 1045- or a 272-nt putative minimal RRE. The gene transfer vectors were modified with the 1045 nt SIV RRE to more closely mimic the remnant HIV-1 RRE containing *env *sequence present in the control vector. Thus, both test and control gene transfer vectors included the 3'*tat/rev *splice acceptor site upstream of the transgene expression cassette.

As a first step, we wished to determine the effect of different Rev-like 'transport' proteins on vector stock production. To this end, vector stocks were produced in 293T cells using various combinations of packaging and gene-transfer vectors encoding EGFP. All transfections received a vesicular stomatitis virus G glycoprotein expression construct (pMD.G), a Tat expression construct (pCMVtat) and a plasmid encoding secreted alkaline phosphatase (SEAP). Each transfection also received a Rev (HIV-1 Rev or SIV Rev) or HTLV-1 Rex expression construct. Virus titers in the supernatants of transfected cells were determined by infection of naïve 293T cells followed by flow cytometry to enumerate GFP+ cells [11]. The titers were adjusted for transfection efficiency by normalizing to the SEAP levels in the vector containing supernatant.

**Figure 2:**
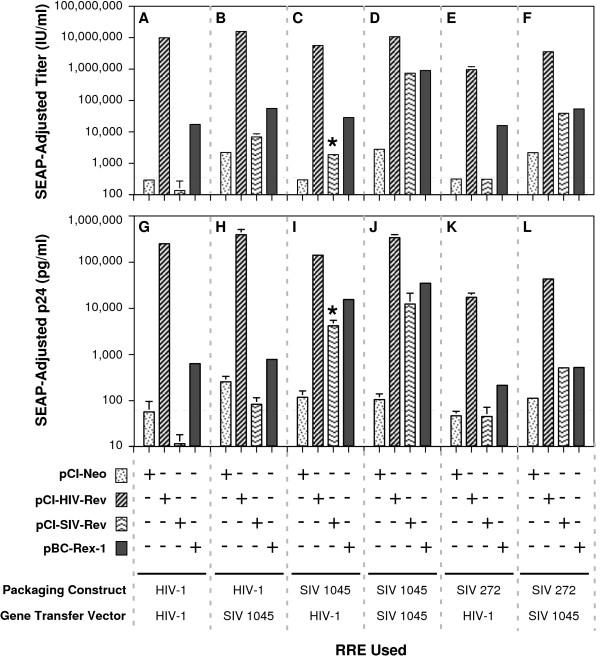
**Effect of Rev and Rex expression constructs on virus stock production by packaging and gene-transfer vectors containing the HIV-1 or SIV RREs**. Vector stocks were produced using various combinations of packaging and gene transfer vectors containing either HIV-1 or SIV RREs as shown. SIV 1045 or SIV 272 refers to the length of SIV RRE present in the packaging construct or gene transfer vector. The effect of expression of Rev-like proteins from HIV-1 (pCI-HIV-Rev), SIV (pCI-SIV-Rev) and HTLV-1 Rex (pBCRex-1) on vector stock production was tested with each of the combinations. A control vector, pCI-Neo, was used in parallel. The mean vector titers, shown in the top panel, were calculated from % GFP positivity determined by flow cytometry. The bottom panel depicts mean p24 levels in the vector containing supernatants. The titers and p24 levels were normalized to SEAP activity present in the vector stock. Error bar = 1 SD. IU: infectious units. '*' denotes relatively high p24 levels with respect to titer (described in greater detail in the text).

The results of vector titer determinations are shown in Figure 2(A–F). The control packaging system (Figure 2A) that used HIV-1 RRE in both packaging and gene transfer vector constructs provided SEAP-adjusted titers of 9.9 ± 0.45 × 10^6 ^infectious units per ml (I.U/ml) in the presence of HIV-1 Rev. Lower titers (1.7 ± 0.07 × 10^4 ^IU/ml) were achieved with HTLV-1 Rex. The SIV Rev was unable to function with the HIV-1 RRE as deduced from the basal vector titers obtained. When the 1045 bp SIV RRE was used in both the packaging and gene transfer vector constructs (Figure 2D), as anticipated, viral titers significantly above basal were obtained with all three 'transport' proteins. Again, highest titers (1.1 ± 0.08 × 10^7^) that were comparable to titers obtained with the control HIV-1 RRE based packaging system were obtained with the HIV-1 Rev. Titers were similar for SIV Rev (7.4 ± 0.2 × 10^5^) and HTLV-1 Rex (9.0 ± 0.7 × 10^5^), but the titers achieved were about an order of magnitude lower. The results were similar for a packaging system that used SIV RRE of 272-nucleotide length in the packaging construct (Figure 2F); however, the titers (3.6 ± 0.09 × 10^6 ^IU/ml) with HIV-1 Rev were lower than for the 1045 nt SIV RRE-based packaging system. When a combination or mixed packaging system was used, i.e. the packaging and gene transfer vectors used HIV-1 RRE for expression of one construct and the SIV RRE for the other construct (Figure 2B, 2C and 2E), higher than basal viral titers were obtained in the presence of HIV-1 Rev or HTLV-1 Rex. The SIV Rev achieved only a marginal increase in titer over that obtained in the absence of any 'transport' protein expression construct. These results suggest that both packaging and gene transfer vector constructs must contain SIV RRE to provide useful titers with SIV Rev. The results also demonstrated that the HIV-1 packaging system with the1045 nt RRE provided titers higher than one with the 272 nt RRE. Finally, the results showed that a packaging system with 1045 nt SIV RRE achieved titers equal to that of the HIV-1 RRE-based one. The results, demonstrating the non-reciprocal nature of interaction of HIV-1 and HIV-2 or SIV Revs with the homologous and heterologous RREs, are consistent with the previous observations of Lewis, et al. [9] and Berchtold et al. [8].

To correlate vector titers to particle production, the supernatants used for infection were tested for HIV-1 p24 by ELISA. The SEAP-adjusted p24 levels are depicted in Figure 2 (panels G through L). The p24 levels showed a very good correspondence to vector titers, with a few notable exceptions. Vector stocks produced with the helper construct pGP/1045 SIV RRE and the gene transfer vector pN-EF1α-EGFP-WPRE in conjunction with pCI-SIV Rev demonstrated relatively high p24 levels (Figure 2 panel I) but low titer (Figure 2 panel C, indicated by an asterisk '*'). This can be explained if SIV Rev functions only with SIV RRE (in the packaging construct) but not with HIV-1 RRE (in the gene transfer vector). Both HIV-1 Rev and SIV Rev functioned less efficiently with the 272 nt SIV RRE in comparison to the 1045 nt SIV RRE (Figure 2, panels E, F, K and L).

### Determination of optimal concentrations of HIV-1 and SIV Rev expression plasmids for use with SIV RRE containing packaging system

**Figure 3:**
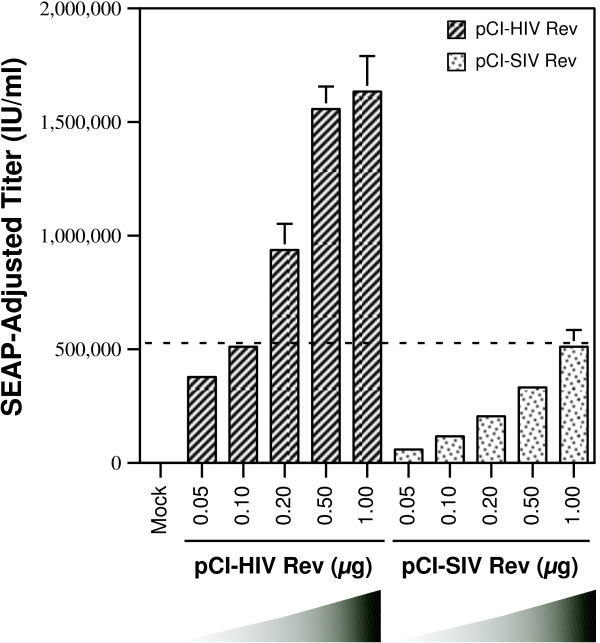
**Determination of optimal amounts of pCI-HIV-1 Rev for production of vector stocks with the SIV RRE-based HIV-1 packaging system**. Vector stocks were produced in 293T cells using pGP/SIV 1045 RRE and pN-EF1α-EGFP-WPRE/SIV RRE with indicated amounts of pCI-HIV Rev or pCI-SIV Rev. The transfections also included pCMVtat, pMD.G and a SEAP expression construct. The vector stocks were used for infection of 293T cells and the resultant SEAP-adjusted vector titers are shown. Error bar = 1 SD.

HIV-1 Rev, in the previous experiment, achieved approximately 10-fold higher titers with the SIV RRE containing packaging system than SIV Rev. One possible interpretation of this result would be that HIV-1 Rev was more efficient than SIV Rev with SIV RRE. An alternative explanation could be that the steady state levels of HIV-1 Rev protein produced were higher than that of SIV Rev. In this case it should be possible to overcome the titer differences with a titration experiment to determine the optimal amounts of each of the Rev expression constructs required with the SIV RRE based packaging system. To this end, the SIV RRE containing packaging system was tested with increasing amounts (0.05 to 1.0 μg) of pCI-HIV Rev or pCI-SIV Rev constructs. The total amount of the 'transport' plasmid used in each transfection was kept constant by using pCI-Neo as a 'filler.' The titers of the resultant vector stocks shown in Figure 3 indicate that pCI-HIV Rev achieved higher titers with the SIV-RRE based packaging system than pCI-SIV Rev with its cognate RRE at all input amounts of each of the Rev expression constructs. To determine if these results could be explained by the steady state levels of the proteins, the lysates of 293T cells transfected with different amounts of pCI-HIV Rev and pCI-SIV Rev were subjected to an immunoblot assay procedure using anti-HA antibody. For the same input amount of Rev expression construct, pCI-HIV Rev showed approximately two-fold higher steady state levels of protein than pCI-SIV Rev (see Additional File 1). At the 0.1 μg amount, pCI-HIV Rev with the SIV RRE containing packaging system provided titers equivalent to that achieved by 1.0 μg of pCI-SIV Rev (indicated by a dashed line in Figure 3). The steady state levels of HIV-1 Rev protein at 0.1 μg was considerably lower than that of SIV Rev at 1.0 μg (see Additional File 1). These data suggest that the increased efficiency of HIV-1 Rev could be partly explained by better Rev expression levels and partly attributed to increased efficiency with SIV RRE. Clearly, additional work is necessary to further probe the reasons for the apparent increased efficiency of HIV-1 Rev over SIV Rev with SIV RRE.

### The SIV RRE-based HIV-1 packaging system is relatively refractory to inhibitory effects of Rev M10

**Figure 4:**
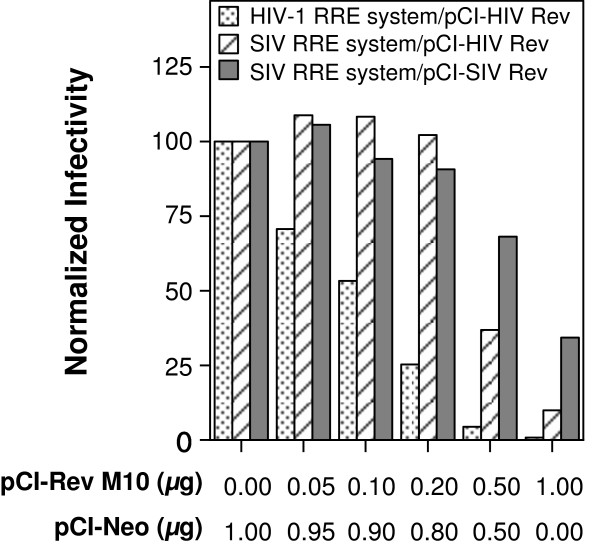
**Effect of increasing amounts of Rev M10-encoding plasmid (pCI-Rev M10) on titer of vector stocks produced with an HIV-1 packaging system containing either HIV-1 or SIV RRE**. The HIV-1 RRE-based packaging system (HIV-1 RRE system) consisted of the packaging plasmid pGP/HIV-1 350 RRE and the gene transfer vector pN-EF1α-EGFP/HIV-1 RRE. The SIV RRE-based packaging system (SIV RRE system) consisted of the packaging plasmid pGP/SIV 1045 RRE and the gene transfer vector pN-EF1α-EGFP/SIV RRE. For production of virus stocks with the SIV RRE-based packaging system either HIV-1 Rev (pCI-HIV Rev) or SIV Rev (pCI-SIV Rev) expression construct was used, as indicated. All transfections also received a VSV-G envelope expression construct (pMD.G) and a HIV-1 Tat (pCMVtat) expression construct. The titers of the vector stocks were determined as described in Materials and Methods. The % of GFP + cells in the absence of pCI-Rev M10 (0 μg) was considered as 100% (Y-axis) for a given packaging system to which other titers obtained at each amount of pCI-Rev M10 were normalized. Increasing amounts of pCI-Rev M10 are depicted on the X-axis. For each transfection, the indicated amount of pCI-Neo was used as a 'filler', to keep the total amount of DNA added at 1.0 μg. The results shown are representative of two independent experiments.

A previous study [8], using a luciferase-based reporter system, showed that the SIV RRE rendered the reporter less susceptible to inhibition by Rev M10. To determine the validity of this observation in the context of gene delivery systems, different amounts (0 μg to 1.0 μg) of an M10 expression construct, pCI-Rev M10, were added during production of vector stock with either the HIV-1 RRE or the SIV RRE-based packaging systems. The total amount of plasmid added was kept constant by using pCI-Neo as a 'filler' plasmid. The pCI-HIV-1 Rev was used for production of vector stock from the HIV-1 RRE-based packaging system. For the SIV RRE-based packaging system, in one set of transfections 0.1 μg of pCI-HIV Rev was used while in another set of transfections, 1.0 μg of pCI-SIV Rev was used. The differing amounts of HIV-1 and SIV Rev expression constructs used with the packaging construct, pGP/SIV 1045 RRE, to ensure comparable vector titers was based on the previous titration experiment (Figure 3). Vector stocks from the different transfections were titrated on Jurkat T-cells and the percentage of cells transduced was determined by flow cytometry. To enable comparison between the different packaging systems, the percentage of EGFP positive cells, obtained in the absence of pCI-Rev M10 for each packaging system, was set at 100%. The results are shown in Figure 4. The Rev M10 protein was found to inhibit the different packaging systems to different degrees. The HIV-1-based packaging system was the most susceptible to inhibition and exhibited a dose-dependant decrease in vector titer, while the SIV RRE-based packaging systems were less susceptible to Rev M10 at the lower doses of pCI-Rev M10. The least susceptible of the SIV RRE based-packaging systems was the one that utilized SIV Rev for expression of HIV-1 helper and gene transfer vector RNAs. Even at the highest amount of Rev M10 expression construct tested (1.0 μg), the titers were reduced by only 2- to 3-fold. This was in contrast to the HIV-1 RRE-based packaging system in which titers decreased by 10- to 100-fold at the highest dosage of M10. Interestingly, the SIV RRE-based packaging system, when used with HIV-1 Rev, also proved to be less susceptible to inhibition than the HIV-1 RRE-based packaging system but more susceptible than the system that used SIV Rev, particularly at high input amounts of pCI-M10. This occurred despite the use of lower amounts of pCI-HIV Rev. A second experiment, using a different HIV-1 vector (pN-GIT72) [7] with the control HIV-1 RRE-based packaging system, provided comparable results (see Additional File 2).

### The SIV RRE-based HIV-1 packaging system provides 34- to 130-fold higher titers than the HIV-1 Rev/RRE-based packaging system when used for packaging Rev M10 encoding gene transfer vectors

We next wished to determine if the SIV RRE-based packaging system would be suitable for delivery of Rev M10 into target cells. To this end, a gene transfer vector, pN-EF1α-EGFP-2A-M10/SIV RRE (Figure 1B), that expressed both Rev M10 and EGFP under control of the EF1α promoter was created. The EGFP and Rev M10 coding sequences were linked in-frame by the 2A cleavage factor sequence from foot and mouth disease virus. For comparison, a vector, pN-EF1α-EGFP-2A-M10/HIV-1 RRE, which expressed EGFP-2A-M10 but had HIV-1 RRE in place of SIV RRE, was used. Other control vectors that expressed only EGFP (Figure 1B) have been alluded to in previous experiments. Different combinations of packaging and gene transfer vectors were used to generate vector stocks. The gene transfer vectors were tested at various input amounts to determine possible impact of the encoded Rev M10 during virus stock production on the vector titer. The vector stocks were produced with either pCI-HIV Rev or pCI-SIV Rev together with other helper constructs, pMD.G and pCMVtat. The titers of the resultant virus stocks were determined using Jurkat T-cells.

The SEAP-adjusted vector titers are summarized in Table 1. Attempts to package an HIV-1 RRE containing Rev M10 encoding vector, pN-EF1α-EGFP-2A-M10/HIV-1 RRE, using the helper construct, pGP/HIV-1 350 RRE resulted in a dose-dependent decrease of 254-, 537- and 862-fold at amounts of 0.75, 1.5 and 3.0 μgs, respectively, in comparison to titers obtained with the control vector that encoded only EGFP, pN-EF1α-EGFP/HIV-1 RRE. When packaging pN-EF1α-EGFP-2A-M10/SIV RRE with pGP/HIV-1 350 RRE, the titer was reduced between 47- and 93-fold. Similarly, when pGP/SIV 1045 RRE was used to package pN-EF1α-EGFP-2A-M10/HIV-1 RRE, the titer was decreased by 119- to 201-fold in comparison to the control vector encoding only EGFP. In contrast, when both the vector encoding Rev M10 and the helper construct contained SIV RRE, the titer drop was only between 6- and 7-fold. This was despite the usage of HIV-1 Rev for packaging the M10 encoding vector. When pCI-SIV Rev was used with pGP/SIV 1045 RRE and pN-EF1α-EGFP-2A-M10/SIV RRE to produce vector stocks, the titer was reduced by 17- and 20-fold. An independent experiment using a subset of the packaging and gene transfer vectors used in this experiment provided similar results (see Additional File 3). Thus an HIV-1 packaging system containing the 1045 nt SIV RRE in both helper and gene tranfer vector construct was superior to the other combinations for delivery of the Rev M10 transgene.

**Table 1:** Efficiency of production of Rev M10 encoding vector stocks using various combinations of packaging and gene transfer vectors containing RRE from HIV-1 or SIVmac239

**Packaging Plasmid/RRE Source**	**Gene Transfer Vector/RRE Source**	**Vector amount used**	**Rev Source**	**SEAP-adjusted Titer (IU/ml) (mean ± SD)**	**Fold difference in Titer^a^**
Mock				0	
pGP/HIV-1	pN-EF1α-EGFP/HIV-1	3.00 μg	HIV-1	2.0 ± 0.2 × 10^5^	1
	pN-EF1α-EGFP-2A-M10/HIV-1	0.75 μg	HIV-1	7.8 ± 3.1 × 10^2^	254
	pN-EF1α-EGFP-2A-M10/HIV-1	1.50 μg	HIV-1	3.7 ± 0.6 × 10^2^	537
	pN-EF1α-EGFP-2A-M10/HIV-1	3.00 μg	HIV-1	2.3 ± 1.0 × 10^2^	862
					
pGP/HIV-1	pN-EF1α-EGFP/SIV	3.00 μg	HIV-1	5.1 ± 0.2 × 10^5^	1
	pN-EF1α-EGFP-2A-M10/SIV	0.75 μg	HIV-1	5.5 ± 2.3 × 10^3^	93
	pN-EF1α-EGFP-2A-M10/SIV	1.50 μg	HIV-1	1.0 ± 0.1 × 10^4^	50
	pN-EF1α-EGFP-2A-M10/SIV	3.00 μg	HIV-1	1.1 ± 0.1 × 10^4^	47
					
pGP/SIV^b^	pN-EF1α-EGFP/HIV-1	3.00 μg	HIV-1	1.1 ± 0.1 × 10^5^	1
	pN-EF1α-EGFP-2A-M10/HIV-1	0.75 μg	HIV-1	9.5 ± 0.3 × 10^2^	119
	pN-EF1α-EGFP-2A-M10/HIV-1	1.50 μg	HIV-1	5.8 ± 2.3 × 10^2^	196
	pN-EF1α-EGFP-2A-M10/HIV-1	3.00 μg	HIV-1	5.7 ± 0.1 × 10^2^	201
					
pGP/SIV	pN-EF1α-EGFP/SIV	3.00 μg	HIV-1	1.8 ± 0.0 × 10^5^	1
	pN-EF1α-EGFP-2A-M10/SIV	0.75 μg	HIV-1	2.7 ± 0.3 × 10^4^	7
	pN-EF1α-EGFP-2A-M10/SIV	1.50 μg	HIV-1	3.2 ± 0.3 × 10^4^	6
	pN-EF1α-EGFP-2A-M10/SIV	3.00 μg	HIV-1	3.0 ± 0.0 × 10^4^	6
					
pGP/SIV	pN-EF1α-EGFP/SIV	3.00 μg	SIV^b^	9.6 ± 1.1 × 10^4^	1
	pN-EF1α-EGFP-2A-M10/SIV	0.75 μg	SIV	4.7 ± 0.1 × 10^3^	20
	pN-EF1α-EGFP-2A-M10/SIV	1.50 μg	SIV	5.1 ± 0.1 × 10^3^	19
	pN-EF1α-EGFP-2A-M10/SIV	3.00 μg	SIV	5.8 ± 0.3 × 10^3^	17

^a ^Fold difference in titer was determined by dividing the titer of the control vector encoding EGFP alone by the titer of the corresponding vector encoding EGFP and Rev M10.

^b^SIV refers to the molecular clone SIVmac239.

### Jurkat T-cells transduced with Rev M10 encoding HIV-1 vectors containing HIV-1 or SIV RRE produce fewer virus particles than cells transduced with control vectors upon challenge with replication defective HIV-1

Jurkat T-cells, separately transduced with each of the four different vectors (pN-EF1α-EGFPE/HIV-1 RRE, pN-EF1α-EGFP-2A-M10/HIV-1 RRE, pN-EF1α-EGFP/SIV RRE, pN-EF1α-EGFP-2A-M10/SIV RRE) in the previous experiment, were sorted to greater than 95% purity and challenged with HIV-1 in single-round infection assays. Since cells containing vector encoding only EGFP exhibited higher levels of EGFP fluorescence than cells containing vector encoding EGFP-2A-M10 (see Additional File 4), the gates for sorting were therefore based on EGFP expression from the EGFP-2A-M10 vector-transduced cells to ensure comparable gene expression levels among the sorted populations.

Each population of sorted cells was either mock-infected or infected with equal volumes of the same virus stock of VSV-G-pseudotyped replication defective molecular clone of HIV-1 (pNL4-3.HSA.R^-^E^-^) challenge virus. After 48 h of infection, the cells were washed six times using complete medium to remove residual virus and placed in culture. The supernatants, harvested from the infected cell cultures after the wash (considered as day 1) and at 72 h intervals post-wash (days 4 and 7), were tested for HIV-1 p24 using a commercial ELISA kit.

**Figure 5:**
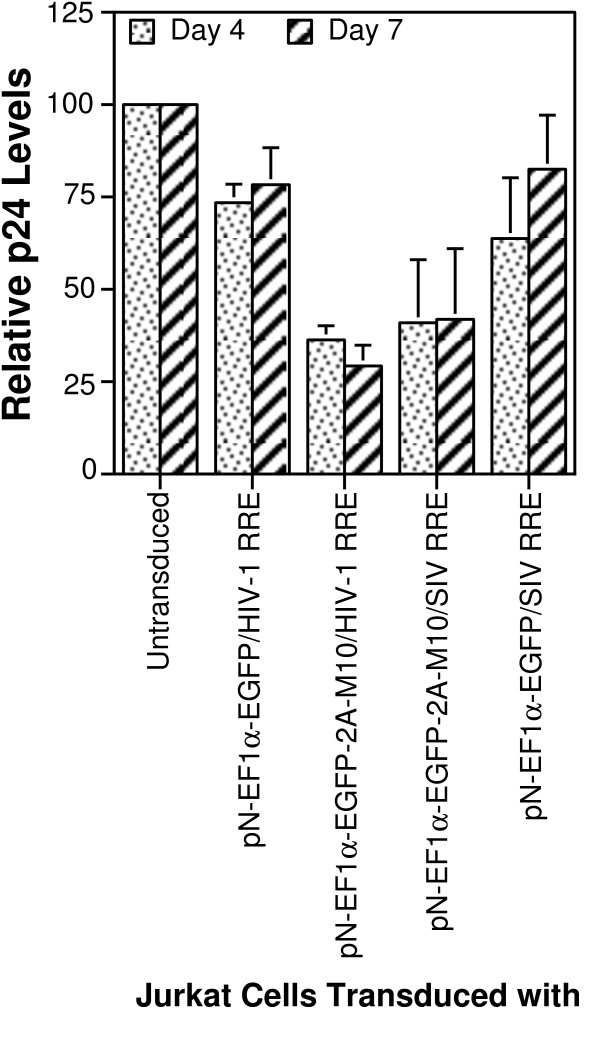
**Virus particle production in Jurkat T-cells transduced with different HIV-1 vectors upon challenge with a replication defective HIV-1**. Jurkat T-cells were separately transduced with each of the indicated vectors (X-axis) and sorted to greater than 95% purity. Each population was either mock-infected or infected with VSV-G pseudotyped pNL4-3.HSA. R^-^E^-^. The supernatants from mock or virus-infected cells were obtained on days 1, 4 and 7 and assayed for HIV-1 p24 capsid protein using a commercial ELISA kit. The Y-axis shows mean p24 levels produced by each of the different cell populations on days 4 and 7 normalized to the p24 produced by infected but untransduced Jurkat cells (which was set at 100%). The results shown are from three independent experiments. Error bar = 1 SD.

The results of ELISA of infected cell culture supernatants are shown in Figure 5 and represent data from three independent experiments. The p24 levels were normalized to that produced in untransduced control Jurkats T-cells infected with the same amount of challenge virus to allow comparison of results obtained in the three independent experiments. The results indicate that the greatest reduction in HIV-1 p24 were seen in supernatants of Jurkat T-cells transduced with M10-encoding vectors (pN-EF1α-EGFP-2A-M10/HIV-1 RRE and pN-EF1α-EGFP-2A-M10/SIV RRE) in comparison to cells transduced with the vectors expressing only EGFP (pN-EF1α-EGFP/HIV-1 RRE or pN-EF1α-EGFP/SIV RRE) (p ≤ 0.05 by using Student's *t*-test). Thus, both the HIV-1 and SIV RRE bearing vectors encoding Rev M10 proved equally effective in diminishing particle production upon challenge with wild-type virus. Interestingly, p24 was also reduced, albeit to less impressive levels, in the supernatant of Jurkat T-cell populations transduced with vectors containing only EGFP (p ≤ 0.05) in comparison to the untranduced control cells. The differences between the different vector transduced cell populations could not be attributed to differences in infection levels since flow cytometry using PE-conjugated antibody to mouse CD24 (heat stable antigen) present in the challenge virus showed comparable levels of infection (see Additional File 5).

## Discussion

There has been a resurgence of interest in evaluating Rev M10 for intracellular immunization in HIV-1 infected patients [2]. However, the usage of HIV-1-based packaging systems to deliver Rev M10 has been particularly problematic due to the inhibitory effect of Rev M10 on vector stock production (Figure 4). Modifications to the HIV-1 packaging system to render it resistant to Rev M10, such as the use of the constitutive transport element of Mason-Pfizer monkey virus [12], would enable its use for anti-HIV-1 gene therapy.

In this study, we describe the use of SIV RRE to replace the HIV-1 RRE in a HIV-1 based-packaging system to achieve the same ends. The results showed that the SIV RRE was able to substitute for the HIV-1 RRE in both packaging as well as the gene transfer vector constructs. The SIV RRE-based packaging systems were found to be not only as efficient as the HIV-1 RRE-based one for production of vector stocks (Figure 2), but also relatively refractory to Rev M10 (Figure 4), despite the use of HIV-1 Rev for production of vector stocks. Our study confirms and extends the earlier study by Berchtold and coworkers [8] who, using a different reporter construct based on expression of luciferase, also showed resistance to Rev M10 of SIV RRE containing construct. To the best of our knowledge, our study is the first to evaluate SIV RRE in the context of an HIV-1-based gene delivery system.

The ability of SIV RRE to render the packaging system relatively resistant to Rev M10 allowed the production of high-titered stocks of vectors encoding Rev M10 (Table 1). When Jurkat T-cells transduced with M10 encoding SIV-RRE containing vectors were challenged with a replication defective HIV-1, in single round infection assays, cells transduced with the Rev M10 encoding vectors produced lower amounts of virus particles than cells transduced with vectors encoding EGFP alone (Figure 5). The differences observed between the Rev M10 expressing cells and control cells, unmodified or expressing EGFP alone, could not be attributed to different levels of infection of the cells since flow cytometry using anti-mouse CD24 antibodies to heat-stable antigen revealed that the percentage of cells infected in the M10 expressing population was similar to the control EGFP expressing cells (Additional File 5). The differences between the different vectors could also not be assigned to variations in the level of Rev M10 or EGFP expression since the sorting was carried out using a narrow window to ensure that comparable levels of EGFP expression was present in the different populations. Moreover, both vectors achieved similar levels of expression of Rev M10 as deduced from EGFP levels since EGFP and Rev M10 expression was linked at the translational level.

Despite the ability of SIV RRE to mitigate the inhibitory effects of Rev M10 during vector stock production, the observation that the SIV RRE containing vector encoding Rev M10 was found to be as efficient as the control Rev M10 expressing vector containing HIV-1 RRE in decreasing HIV-1 particle production (Figure 4) in transduced target cells can be explained as follows. The presence of constitutively expressed Rev M10 in the target cell, due to gene modification, would ensure interference with the function of wild-type Rev produced from the challenge virus, even at the earliest time points. This would then prevent significant accumulation of full-length viral RNA that encodes Gag/Pol or the vector RNA containing SIV RRE. Thus, the concentration of SIV RRE containing transcript in the gene-modified Jurkat T-cell is likely to be too low to obtund Rev M10 function.

In addition to the use of the SIV RRE based packaging system for delivery of Rev M10, one could possibly use such a system for targeting the HIV-1 envelope sequence employing RNAi approaches. Employing distinct RNA transport elements for expression of helper and gene transfer vector RNA can reduce the risk of recombination between the packaging and gene transfer vector constructs during vector stock production [13,14]. Such packaging systems can be used for delivery of any transgene of interest. Here we have demonstrated that the SIV RRE can replace HIV-1 RRE in either the packaging or gene transfer vector with no loss of titer.

An alternative approach to decreasing recombination frequency between components of packaging systems is by using hybrid packaging systems consisting of helper and gene transfer constructs derived in their entirety from viruses with low sequence homology, such as SIV (or HIV-2) and HIV-1 [15,16]. The major concern in the case of the hybrid packaging systems is the low efficiency of encapsidation of the heterologus vector RNA [17,18] in comparison to the homologus vector RNA. In contrast to those studies, HIV-1 packaging systems that utilize only the SIV RRE of the different viruses are not likely to have such drawbacks. However, a direct comparison of the different packaging systems is necessary to determine the suitability of different packaging systems for specific therapeutic applications.

It was previously hypothesized that the Rev M10 protein inhibits wild-type Rev function by formation of mixed-multimers with wild-type Rev protein [19,20]. The findings in this study, and that of Berchold and coworkers [8], appear to challenge that hypothesis since the mere presence of SIV RRE in the producer cell seemed to obtund the inhibitory effect of Rev M10 on wild type Rev. The reasons for this are not clear but one possible mechanism could be a 'squelching' effect of Rev M10 by SIV RRE during virus stock production. An alternative hypothesis is that HIV-1 Rev bound to SIV RRE may be able to access the nucleo-cytoplasmic transport pathway downstream of the Rev M10 effect or the SIV RRE-HIV-1 Rev complex may be able to use a different pathway not amenable to inhibition by Rev M10. Further investigations should shed light on the different possibilities.

## Conclusion

The present study demonstrated that an HIV-1-based packaging system containing only the RRE sequence from SIV can be used for efficient delivery of Rev M10 into HIV-1 susceptible cells to achieve intracellular immunization. Furthermore, the studies showed that SIV RRE could be used in the context of a reciprocal or combination packaging system to improve its safety without compromising vector titers.

## Methods

### Plasmid constructs

#### Packaging constructs

The packaging constructs (Figure 1A) contain the *gag/pol *coding region, nt 711 to nt 5122, from the HIV-1 molecular clone, pNL4-3 [GenBank:M19921], with a deletion of the encapsidation signal between nt 751 and nt 779. The viral coding sequence was inserted into pCDNA3 (Invitrogen, Carlsbad, CA) downstream of the human cytomegalovirus immediate-early promoter and upstream of the bovine growth hormone polyadenylylation sequence. The RNA transport sequences were inserted between the *gag/pol *coding sequence and the polyadenylylation signal. The construct pGP/HIV-1 350 RRE contains a 350 nt HIV-1 RRE (nt 7701 to nt 8050 of pNL4-3). The construct pGP/SIV 1045 RRE contains a 1045 nt SIV RRE (nt 8328 to nt 9372 of SIVmac239; [GenBank:M33262]) while pGP/SIV 272 RRE contains a 272 nt SIV RRE (nt 8456 to nt 8727 of SIVmac239). The SIV RRE sequences were derived from the plasmid construct pTR170 [21].

#### Gene-transfer vectors

The gene-transfer vectors (Figure 1B) are similar to the previously described pN-EF1α-MGMT-WPRE vector [22]. The vector pN-EF1α-EGFP/HIV-1 RRE was derived from the molecular clone pNL4-3 and has a deletion between proximal (nt 1247) and distal (nt 6738) NsiI sites of pNL4-3. The remnant portion of the HIV-1 *env *contains the RRE. The vector has an engineered frame-shift (FS) mutation in *gag *[6] and the central polypurine tract and central termination sequences (CPPT/CTS) to improve gene-transfer efficiency [23-25]. The transgene expression cassette, positioned between the BamHI site in the second coding exon of Rev that overlaps the 3' end of *env *and the XhoI site in *nef*, consists of human elongation factor 1 alpha (EF1α) promoter driving enhanced green fluorescent protein (EGFP). The woodchuck post-transcriptional regulatory element (WPRE) [26] was placed downstream of the EGFP coding sequence. To create the SIV RRE containing vector pN-EF1α-EGFP/SIV RRE, a 1045 nt SIV RRE (described above for pGP/SIV 1045 RRE) was inserted between BsaBI and EcoRI sites of pN-EF1α-EGFP/HIV-1 RRE, effectively replacing the HIV-1 RRE with that of SIV. The vectors pN-EF1α-EGFP-2A-M10/HIV-1 RRE and p-EF1α-EGFP-2A-M10/SIV RRE are identical to the above-described vectors but express both EGFP and Rev M10 instead of EGFP alone. The EGFP and Rev M10 coding sequences were linked in-frame by the foot and mouth disease virus 2A cleavage factor sequence. Inclusion of the 2A sequence in-frame results in the cleavage and release of EGFP-2A and Rev M10 proteins from the engineered polyprotein and ensures equimolar expression of both transgenes [27,28].

#### pCI-HIV-Rev and pCI-Rev M10

These contain the HIV-1 Rev coding sequence amplified from pCMVRev (corresponds to nt 970 to nt 1320 in HIVPCV12; [GenBank:M11840]) with an added hemagglutinin (HA) epitope tag (MYPYDVPDYA) at the N-terminus and inserted into pCI-Neo (Promega Corp., Madison, WI) between the human cytomegalovirus immediate early promoter and polyadenylylation signal of SV40 virus. A synthetic intron is present upstream of the Rev coding sequence. pCI-Rev M10 is identical to pCI-HIV-Rev but contains the classic mutation in the nuclear export sequence (LQLPPLERLTLD) of HIV-1 Rev in which residues LE (CTTGAG) were changed to DL (GATCTC) [10].

#### pCI-SIV Rev

This plasmid contains the Rev coding sequence amplified from p239SpE3' [29] which contains the 3' half of SIVmac239. The SIV Rev corresponds to nt 6784 to nt 6853 (first coding exon) and nt 9062 to nt 9315 (second coding exon) of SIVmac239 joined in-frame using splicing by overlap extension (SOE) PCR [30,31]. An N-terminal HA epitope tag was engineered in the same manner as for pCI-HIV-Rev. The amplified sequence was inserted into pCI-Neo as described above for pCI-HIV-Rev.

#### Other plasmid constructs

Constructs pCMVTat (expresses HIV-1 Tat), pCMVRev, and pBC-Rex-1 (expresses HTLV-1 Rex) [9] were kindly made available by Drs. David Rekosh and Marie-Louise Hammaskjöld (University of Virginia, Chalottesville, VA). Construct pMD.G (expresses vesicular stomatitis virus G glycoprotein) was a generous gift of Dr. Didier Trono (University of Geneva Medical School, Geneva, Switzerland). The replication defective challenge virus, pNL4-3.HSA.R^-^E^-^, was kindly provided by Dr. Nathaniel Landau through the NIH AIDS Research and Reference Reagent Program, Division of AIDS, NIAID, NIH. pNL4-3.HSA.R^-^E clone does not express HIV-1 Env, Nef or Vpr. It contains the coding sequence for mouse heat-stable antigen (HSA) in place of Nef that allows enumeration of virus titer by flow cytometry of infected cells following staining with fluorochrome-conjugated anti-mouse CD24 antibody.

### Transfections

Transfections were carried out by the CaPO_4 _procedure as detailed elsewhere [11]. Briefly, 293T cells (0.75 × 10^5 ^cells), seeded in 6-well plates cells on the previous day, were transfected with the indicated plasmids at the stated amounts. Transfections also received a construct encoding secreted alkaline phosphatase (SEAP) and/or pEGFP-N1 (Clontech, Mountain View, CA) to compare transfection efficiencies. Assays for SEAP in culture supernatants were done using a commercial kit (Phospha-Light System, Applied Biosystems, Bedford, MA) as per the manufacturer's instructions. For transfections receiving pEGFP-N1, efficiency of transfection was estimated by visual inspection under fluorescence microscopy. pEGFP-N1 was not used for those transfections that included a gene transfer vector encoding EGFP which also allowed visual estimation of transfection efficiency.

### Immunoblot Assay

This was done as previously described [32]. Briefly, proteins in cell lysates were resolved by SDS-PAGE (12 to 15% acrylamide concentration) and transferred to Immobilon-P membranes. The membranes were probed with indicated antibodies or antibody-horse-radish-peroxidase conjugates. The bound conjugates were visualized using a chemiluminiscent substrate (Lumi-Light Western Blotting Substrate, Roche Molecular Biochemicals, Mannheim, Germany) and X-ray films.

### Production of vector stocks

The 293T cells were transfected with 1.5 μg of packaging plasmid, 3.0 μg of gene-transfer vector, 0.2 μg of VSV-G expression construct (pMD.G), 0.2 μg of pCMVtat, and indicated amounts of pCI-HIV-Rev or other regulatory protein expression construct. Other expression constructs were used as necessary and as described in the Results and Discussion section. The following day the medium was replaced with fresh medium. Virus-containing medium was harvested 48 h after the first medium change. Cellular debris was removed by centrifugation at 1,428 × g (R_max_), and the resultant virus stock was either used immediately for infection or saved in aliquots at -80°C.

### Titration of vector stock

Naïve 293T or Jurkat T-cells (2 × 10^5 ^cells) were infected with aliquots of virus stock in the presence of polybrene (10 μg/ml) in 0.5 ml of cell culture medium. Two volumes of fresh medium were added next day to dilute the polybrene. The cells were maintained for 48 to 72 hours prior to harvest and fixation with freshly prepared 4% paraformaldehyde. Since all gene transfer vectors encoded EGFP, titers (infectious units/ml) were calculated from the percentage of GFP+ cells as determined by flow cytometry [11] and using the formula: { [(% GFP+ cells ÷ 100) × total number of target cells at the time of infection] ÷ volume used for infection} × 1000.}

### Creation of pooled populations of Jurkat cells expressing EGFP or EGFP-2A-M10

Jurkat T-cells were separately transduced with HIV-1 vectors encoding either EGFP alone or EGFP and Rev M10. Transduced cells were sorted to a purity of greater than 95% using a BD FACSAria flow cytometer (BD Biosciences, San Jose, CA).

### Challenge experiments

Jurkat cells, 2.5 × 10^5 ^cells in 0.5 ml of medium, expressing EGFP or EGFP and Rev M10, were infected with the replication defective HIV-1 molecular clone pNL4-3.HSA.R^-^E^- ^[33,34] in the presence of 10 μg/ml of polybrene. This clone does not express HIV-1 Env, Nef or Vpr and is therefore replication defective. It contains the coding sequence for mouse heat-stable antigen (HSA) in place of Nef that allows enumeration of virus titer by flow cytometry of infected cells following staining with fluorochrome-conjugated anti-mouse CD24 antibody. Since the virus does not express HIV-1 envelope glycoprotein, the virus stocks were prepared by cotransfection with a VSV-G expression plasmid (pMD.G). The day after infection an additional one ml of complete medium was added. After a further 48 hr, the Jurkat cells were washed 6 times in complete medium to remove residual input virus before returning the cells to the incubator in a new 24-well tissue culture plate. The cells were split at a ratio of 1:5 or 1:10 after another 72 h (day 4). The supernatants were collected immediately after the cells were washed (considered day 1) and at 72 h intervals thereafter (days 4 and 7) and assayed for HIV-1 p24 using a commercial ELISA kit (Perkin-Elmer, Boston, MA).

## List of abbreviations

bp: base-pair; CPPT/CTS: Central polypurine tract/central termination sequence; Ef1α: Elongation factor 1 alpha; EGFP: Enhanced green fluorescent protein; HIV-1: Human immunodeficiency virus type 1; SEAP: Secreted alkaline phosphatase; SIV: Simian immunodeficiency virus; nt: nucleotide; RRE: Rev-response element; HSA: Heat-stable antigen; ELISA: Enzyme-linked immunosorbent assay.

## Competing interests

The author declares that he has no competing interests.

## Authors' contributions

The experiments described here were conceived and performed by the author with technical assistance of Ms. Margo Camel in some of the experiments.

## Supplementary Material

Additional file 1 **Immunoblot of cell lystes of 293T cells tranfected with pCI-HIV Rev or pCI-SIV Rev**. 293T cells were transfected with indicated amounts of pCI-HIV Rev or pCI-SIV Rev. The total amount of plasmid added was kept constant by adding pCI-Neo as filler to achieve 1 μg per transfection. The cells were harvested 72 h post-transfection and equal volumes of cell lysates were resolved on polyacrylamide gels, transferred to PVDF membranes and probed with horse-radish peroxidase conjugated antibody directed to the HA epitope. Bound antibodies were visualized using a chemiluminescent substrate and X-ray films. Click here for file

Additional file 2 **Effect of increasing amounts of Rev M10-encoding plasmid (pCI-Rev M10) on titer of vector stocks produced with an HIV-1 packaging system containing either HIV-1 or SIV RRE**. The experiment was conducted as for Figure 4 but using a different gene transfer vector, pN-GIT72, [7] for the HIV-1 RRE-based packaging system. Click here for file

Additional file 3 **Supplemental Table**. Efficiency of production of Rev M10 encoding vector stocks using various combinations of packaging and gene transfer vectors containing RRE from HIV-1 or SIVmac239. Click here for file

Additional file 4 **Flow cytometry profiles of Jurkat T-cells transduced with HIV-1 vectors encoding EGFP or EGFP-2A-Rev M10**. The packaging constructs used for preparation of the vector stocks are shown on the Y-axis while the gene transfer vectors are shown on the X-axis. The attributes of the gene transfer vectors, such as the presence of HIV-1 or SIV RRE, the transgene expressed (EGFP or EGFP-2A-M10) are indicated. GFP expression is depicted along the X-axis and forward scatter (FSC) is indicated along the Y-axis. The percentage and the geometric mean of fluorescence intensity (GMFI) of the EGFP positive populations are shown. Vector stocks for transductions shown in panels A through H were produced using pCI-HIV-Rev while those for transductions in J and K used pCI-SIV-Rev. Representative data from two independent experiments. Click here for file

Additional file 5 **Flow cytometry profiles of Jurkat T-cells challenged with pNL4 R^-^E^-^HSA+ virus**. Each population of Jurkat T-cells was stained with anti-mouse CD24 antibody conjugated with phycoerythrin (PE), washed and fixed with 4% paraformaldehyde before analysis by flow cytometry. GFP expression is shown along the X-axis while staining for mouse HSA with PE-conjugated anti-CD24 antibody is shown along the Y-axis. Both mock-infected and challenge virus-infected cells are shown. The vectors present in the different populations are indicated as follows: EGFP/HIV-1 RRE = pN-EF1α-EGFP-WPRE/HIV-1 RRE; EGFP/SIV RRE = pN-EF1α-EGFP-WPRE/SIV RRE; EGFP-2A-M10/HIV-1 RRE = pN-EF1α-EGFP-2A-M10-WPRE/HIV-1 RRE; EGFP-2A-M10/SIV RRE = pN-EF1α-EGFP-2A-M10-WPRE/SIV RRE. The percentage of cells positive for HSA in EGFP negative (upper left) and positive (upper right) populations are indicated. The geometric means of fluorescence intensity (GMFI) of mock-infected cells are shown. Representative data from two independent experiments. Click here for file
