# Supplementary material for: Substitution of the Rev-response element in an HIV-1-based gene delivery system with that of SIVmac239 allows efficient delivery of Rev M10 into T-lymphocytes
Source: AIDS Res Ther. 2008 Jun 5;5:11. doi: 10.1186/1742-6405-5-11 (PMC2438438; doi:10.1186/1742-6405-5-11)
Supplement: Additional file 3 — Supplemental Table. Efficiency of production of Rev M10 encoding vector stocks using various combinations of packaging and gene transfer vectors containing RRE from HIV-1 or SIVmac239. [file 1742-6405-5-11-S3.doc]

Table. Efficiency of production of Rev M10 encoding vector stocks using various combinations of Packaging and gene transfer vectors containing RRE from HIV-1 or SIVmac239

| **Packaging Plasmid /RRE Source** | **Gene Transfer Vector /RRE Source** | **Vector amount used** | **Rev Source** | **SEAP-adjusted Titer (IU/ml)**  **(mean ± SD)** | **Fold difference in Titer^a^** |
| --- | --- | --- | --- | --- | --- |
| Mock |  |  |  |  |  |
| pGP/HIV-1 | pN-Ef1a-EGFP /HIV-1 | 3.00 µg | HIV-1 | 4.4 ± 0.03 x 10^5^ | 1 |
|  | pN-Ef1a-EGFP-2A-M10 /HIV-1 | 0.75 µg | HIV-1 | 1.9± 1.7 x 10^3^ | 231 |
|  | pN-Ef1a-EGFP-2A-M10 /HIV-1 | 1.50 µg | HIV-1 | 1.4±0.49 x 10^3^ | 314 |
|  | pN-Ef1a-EGFP-2A-M10 /HIV-1 | 3.00 µg | HIV-1 | 2.7±11 x10^2^ | 1629 |
|  |  |  |  |  |  |
| pGP/HIV-1 | pN-Ef1a-EGFP/SIV | 3.00 µg | HIV-1 | ND^c^ |  |
|  | pN-Ef1a-EGFP-2A-M10 /SIV | 0.75 µg | HIV-1 | 4.6± 2.1 x 10^4^ |  |
|  | pN-Ef1a-EGFP-2A-M10 /SIV | 1.50 µg | HIV-1 | 5.3±0.04x10^4^ |  |
|  | pN-Ef1a-EGFP-2A-M10 /SIV | 3.00 µg | HIV-1 | 3.1±0.15x10^4^ |  |
|  |  |  |  |  |  |
| pGP/SIV | pN-Ef1a-EGFP/SIV | 3.00 µg | HIV-1 | 6.6±1.5x10^5^ | 1 |
|  | pN-Ef1a-EGFP-2A-M10 /SIV | 0.75 µg | HIV-1 | 9.9±0.53x10^4^ | 6.7 |
|  | pN-Ef1a-EGFP-2A-M10 /SIV | 1.50 µg | HIV-1 | 1.3±0.12x10^5^ | 5 |
|  | pN-Ef1a-EGFP-2A-M10 /SIV | 3.00 µg | HIV-1 | 1.3±0.11x10^5^ | 5 |
|  |  |  |  |  |  |
| pGP/SIV | pN-Ef1a-EGFP/SIV | 3.00 µg | SIV^b^ | 3.5±0.3 x10^5^ | 1 |
|  | pN-Ef1a-EGFP-2A-M10 /SIV | 0.75 µg | SIV | 1.6±0.06 x10^4^ | 21 |
|  | pN-Ef1a-EGFP-2A-M10 /SIV | 1.50 µg | SIV | 2.0±0.07x10^4^ | 17.5 |
|  | pN-Ef1a-EGFP-2A-M10 /SIV | 3.00 µg | SIV | 1.9±0.05x10^4^ | 18.4 |

^a^ Fold difference in titer was determined by dividing the titer of the control vector encoding EGFP alone by the titer of the corresponding vector encoding EGFP and Rev M10.

^b^ SIV refers to the molecular clone SIVmac239.

^c^ Not done
